# Opial inequality in *q*-calculus

**DOI:** 10.1186/s13660-018-1928-z

**Published:** 2018-12-14

**Authors:** Tatjana Z. Mirković, Slobodan B. Tričković, Miomir S. Stanković

**Affiliations:** 1 College of Applied Professional Studies, Vranje, Serbia; 2 Department of Mathematics, Faculty of Civil Engineering, University of Niš, Niš, Serbia; 3 Mathematical Institute, Serbian Academy of Sciences and Arts, Beograd, Serbia

**Keywords:** 81P68, 26D10, *q*-derivative, *q*-integral, Opial’s inequality

## Abstract

In this article we give *q*-analogs of the Opial inequality for *q*-decreasing functions. Using a closed form of the restricted *q*-integral (see Gauchman in Comput. Math. Appl. 47:281–300, 2004), we establish a new integral inequality of the *q*-Opial type.

## Introduction

In 1960, Opial [10] established the following important integral inequality.

### Theorem 1.1

*Let* $f\in C^{1}[0,h]$, *where* $f(0)=f(h)=0$ *and* $f(t)>0$ *for* $t\in(0,h)$. *Then*

1 $$ \int_{0}^{h} \bigl\vert f(x)f'(x) \bigr\vert \,dx \le\frac{h}{4} \int_{0}^{h} { \bigl(f'(x) \bigr)^{2} \,dx }. $$

*The constant* $\frac{h}{4}$ *is the best possible*.

Integral inequalities of the form (1) have an interest in itself, and also have important applications in the theory of ordinary differential equations and boundary value problems (see [1, 2, 4]). In the years thereafter, numerous generalizations, extensions and variations of the Opial inequality have appeared (see [12, 14]). The one containing fractional derivatives is investigated as well (see [3, 5]).

In the continuous case, the Opial inequity, in its modified form, states that if $f(x)$ is an absolutely continuous function with $f(a) = 0$, and $f' \in L^{2} (a,b)$ where *a* and *b* are finite, then

$$\int_{a}^{b} { \bigl\vert f(x)f'(x) \bigr\vert \,dx } \le\frac{1}{2}(b - a)^{2} \int_{a}^{b} { \bigl(f'(x) \bigr)^{2} \,dx }, $$

with equality attained only if $f(x) = c(x - a)$.

In a recent paper [14], Yang proved the following generalization of the Opial inequality.

### Theorem 1.2

*If* $f(x)$ *is absolutely continuous on* $[a,b]$ *with* $f(a) = 0$, *and if* $p,\,q \geq1$, *then*

$$\int_{a}^{b} { \bigl\vert f(x) \bigr\vert ^{p} \bigl\vert f'(x) \bigr\vert ^{q} \,dx } \le\frac{q}{p + q}(b - a)^{p} \int_{a}^{b} { \bigl\vert f'(x) \bigr\vert ^{p + q} \,dx }. $$

## Preliminaries

Here we present necessary definitions and facts from the *q*-calculus. We follow the terminology and notations used in the books [8, 9, 11, 13]. In what follows, *q* is a real number satisfying $0 < q < 1$, and *q*-natural number is defined by

$$[n]_{q} = \frac{1 - q^{n}}{1 - q} = q^{n - 1}+\cdots+q+1,\dots,\quad n\in\mathbb {N}. $$

### Definition 2.1

Let *f* be a function defined on an interval $(a,b)\subset \mathbb {R}$, so that $qx\in(a,b)$ for all $x\in(a,b)$. For $0 < q <1$, we define the *q*-derivative as

2 $$ (D_{q} f) (x) = \frac{f(x) - f(qx)}{x - qx},\quad x\ne0;\qquad D_{q} f(0)=\lim_{x \to0}D_{q} f(x). $$

In the paper [7], Jackson defined *q*-integral, which in the *q*-calculus bears his name.

### Definition 2.2

The *q*-integral on $[0,a]$ is

$$\int_{0}^{a} f(x)\,d_{q} x = a(1 - q)\sum _{j = 0}^{\infty}q^{j} f \bigl(aq^{j} \bigr). $$

On this basis, in the same paper, Jackson defined an integral on $[a,b]$:

3 $$ \int_{a}^{b} f(x)\,d_{q} x = \int_{0}^{b} f(x)\,d_{q} x - \int_{0}^{a} f(x)\,d_{q} x. $$

For a positive integer *n* and $a=bq^{n}$, using the left-hand side integral of (3), in the paper [6], Gauchman introduced the *q*-restricted integral

4 $$ \int_{a}^{b} f(x)\,d_{q} x= \int_{bq^{n}}^{b} f(x)\,d_{q} x=b(1 -q)\sum _{j=0}^{n-1}q^{j} f \bigl(q^{j} b \bigr).$$

### Definition 2.3

The real function *f* defined on $[a,b]$ is called *q*-increasing (*q*-decreasing) on $[a,b]$ if $f(qx)\le f(x)$ ($f(qx)\ge f(x)$) for $x, qx \in[a,b]$. It is easy to see that if the function *f* is increasing (decreasing), then it is *q*-increasing (*q*-decreasing) too.

## Results and discussions

Our main results are contained in three theorems.

### Theorem 3.1

*Let* $f\in C^{1}[0,1]$ *be* *q*-*decreasing function with* $f(bq^{0})=0$. *Then*, *for any* $p \ge0$,

5 $$\begin{aligned} \int_{a}^{b} \bigl\vert D_{q} f(x) \bigr\vert \bigl\vert f(x) \bigr\vert ^{p} \,d_{q} x \le(b - a)^{p} \int_{a}^{b} \bigl\vert D_{q} f(x) \bigr\vert ^{p + 1}\, d_{q} x. \end{aligned}$$

### Proof

Using Definition 2.1 and (4), we have

$$\begin{aligned} \int_{a}^{b} \bigl\vert D_{q} f(x) \bigr\vert \bigl\vert f(x) \bigr\vert ^{p} \,d_{q} x&= \int_{bq^{n}}^{b} \biggl\vert \frac{f(x) - f(qx)}{x - qx} \biggr\vert \bigl\vert f(x) \bigr\vert ^{p} \,d_{q} x \\ &=b ( {1 - q} )\sum_{j = 0}^{n - 1} q^{j} \biggl\vert \frac{f(bq^{j}) - f(bq^{j + 1}}{bq^{j} - bq^{j + 1} } \biggr\vert \bigl\vert f \bigl(bq^{j} \bigr) \bigr\vert ^{p} \\ &=\sum_{j = 0}^{n - 1} { \bigl\vert f \bigl(bq^{j} \bigr) - f \bigl(bq^{j + 1} \bigr) \bigr\vert } \bigl\vert f \bigl(bq^{j} \bigr) \bigr\vert ^{p} \\ &\le \bigl\vert f \bigl(bq^{n} \bigr) \bigr\vert ^{p} \sum_{j = 0}^{n - 1} \bigl\vert f \bigl(bq^{j} \bigr) - f \bigl(bq^{j + 1} \bigr) \bigr\vert . \end{aligned} $$

In view of $f(bq^{n})=\sum_{j=0}^{n-1}{f(bq^{j+1})-f(bq^{j})}$ and Hölder’s inequality, we obtain

$$\begin{aligned} &\Biggl\vert \sum_{j = 0}^{n - 1} {f \bigl(bq^{j + 1}\bigr) - f\bigl(bq^{j}\bigr)} \Biggr\vert ^{p} \sum_{j = 0}^{n - 1} { \bigl\vert f\bigl(bq^{j}\bigr) - f\bigl(bq^{j + 1}\bigr) \bigr\vert } \\ & \quad\le\Biggl(\sum_{j = 0}^{n - 1} { \bigl\vert f\bigl(bq^{j}\bigr) - f\bigl( bq^{j + 1}\bigr) \bigr\vert }\Biggr)^{p} \sum_{j = 0}^{n - 1} { \bigl\vert f\bigl(bq^{j}\bigr) - f\bigl(bq^{j + 1}\bigr) \bigr\vert } \\ & \quad= \Biggl(\sum_{j = 0}^{n - 1} { \bigl\vert f\bigl(bq^{j}\bigr) - f\bigl(bq^{j + 1}\bigr) \bigr\vert }\Biggr)^{p + 1} \le n^{p} \sum _{j = 0}^{n - 1} { \bigl\vert f\bigl(bq^{j} \bigr) - f\bigl(bq^{j + 1}\bigr) \bigr\vert ^{p + 1} }. \end{aligned}$$

By elementary calculations, we easily transform the right-hand side of the last inequality into

$$n^{p} b^{p} (1-q)^{p} \sum _{j = 0}^{n-1} \bigl(q^{j} \bigr)^{p} \frac{ \vert f(bq^{j}) - f(bq^{j + 1}) \vert ^{p + 1} }{ \vert bq^{j} - bq^{j + 1} \vert ^{p}}. $$

However, because of $0< q<1$, we have

$$\sum_{j = 0}^{n - 1} \bigl(q^{j} \bigr)^{p} \frac{ \vert f(bq^{j}) - f(bq^{j + 1}) \vert ^{p + 1}}{ \vert bq^{j} - bq^{j + 1} \vert ^{p} }\le\sum_{j=0}^{n-1} \frac{ \vert f(bq^{j}) - f(bq^{j + 1}) \vert ^{p + 1}}{ \vert bq^{j} - bq^{j + 1} \vert ^{p} }, $$

meaning that

$$n^{p} b^{p} (1-q)^{p} \sum _{j = 0}^{n-1} \bigl(q^{j} \bigr)^{p} \frac{ \vert f(bq^{j}) - f(bq^{j + 1}) \vert ^{p + 1} }{ \vert bq^{j} - bq^{j + 1} \vert ^{p}}\le-n^{p} b^{p}(1 - q)^{p} \int_{b}^{bq^{n}} { \bigl\vert D_{q} f(x) \bigr\vert ^{p + 1} \,d_{q} x}. $$

Since $n\ge[n]_{q}=\frac{1-q^{n}}{1-q}$, we have $- n^{p} (1 - q)^{p} \le - (1 - q^{n})^{p}$, and we arrive at the inequality

$$\int_{a}^{b} { \bigl\vert D_{q} f(x) \bigr\vert \bigl\vert f(x) \bigr\vert ^{p} \,d_{q} x} \le- b^{p} \bigl(1 - q^{n} \bigr)^{p} \int_{b}^{bq^{n} } { \bigl\vert D_{q} f(x) \bigr\vert ^{p + 1}\, d_{q} x}. $$

After interchanging the boundaries in the right-hand side integral, and replacing $bq^{n}$ with *a*, we find

$$b^{p} \bigl(1 - q^{n} \bigr)^{p} \int_{bq^{n}}^{b} \bigl\vert D_{q} f(x) \bigr\vert ^{p + 1} \,d_{q} x =(b - a)^{p} \int_{a}^{b} \bigl\vert D_{q} f(x) \bigr\vert ^{p + 1}\, d_{q} x, $$

which proves the theorem. □

### Remark 3.2

In particular, by taking $p = 1$, the inequality (9) in Theorem 1 reduces to the following Opial inequality in *q*-calculus:

$$\begin{aligned} \int_{a}^{b} { \bigl\vert D_{q} f(x) \bigr\vert \bigl\vert f(x) \bigr\vert \,d_{q} x} \le(b - a) \int_{a }^{b} { \bigl\vert D_{q} f(x) \bigr\vert ^{2} \,d_{q} x}. \end{aligned}$$

The following theorems are concerned with *q*-monotonic functions.

### Theorem 3.3

*If* $f(x)$ *and* $g(x)$ *are absolutely continuous* *q*-*decreasing functions on* $(a,b)$ *and* $f(bq^{0})=0$ *and* $g(bq^{0})=0$, *then*

6 $$\begin{aligned} \int_{a}^{b} { \bigl[ f(x)D_{q} g(x) + g(qx)D_{q} f(x) \bigr]\,d_{q} x} \le \frac{{a - b}}{2} \int_{a}^{b} { \bigl[ \bigl(D_{q} f(x) \bigr)^{2} + \bigl(D_{q} g(x) \bigr)^{2} \bigr]\,d_{q} x}. \end{aligned}$$

### Proof

Replacing (2) in the integral

$$\int_{a}^{b} \bigl[f(x)D_{q} g(x)+ g(qx)D_{q} f(x) \bigr]\,d_{q} x, $$

we obtain

$$\int_{bq^{n}}^{b} \biggl[f(x)\frac{g(x) -g(qx)}{x - qx}+g(qx) \frac{f(x) - f(qx)}{x - qx} \biggr]\,d_{q} x, $$

whence, using the Gauchman *q*-restricted integral, we have

$$\begin{aligned} & b(1 - q) \Biggl(\sum_{j = 0}^{n - 1} {q^{j} f \bigl(bq^{j} \bigr)\frac{g(bq^{j}) - g(bq^{j + 1})}{bq^{j} - bq^{j + 1}}} +\sum _{j = 0}^{n - 1} {q^{j} g \bigl(bq^{j + 1} \bigr)\frac{f(bq^{j}) - f(bq^{j + 1})}{bq^{j} - bq^{j + 1}}} \Biggr) \\ &\quad =\sum_{j=0}^{n-1} \bigl[f \bigl(bq^{j} \bigr) \bigl(g \bigl(bq^{j} \bigr)-g \bigl(bq^{j+1} \bigr) \bigr)+g \bigl(bq^{j+1} \bigr) \bigl(f \bigl(bq^{j} \bigr)-f \bigl(bq^{j+1} \bigr) \bigr) \bigr]. \end{aligned}$$

Denoting $\Delta f(bq^{j})=f(bq^{j + 1})-f(bq^{j})$ and $\Delta g(bq^{j})=g(bq^{j+1})-g(bq^{j})$, we can rewrite the last sum in the form of $\sum_{j = 0}^{n - 1} [f(bq^{j})\Delta g(bq^{j}) + g(bq^{j + 1})\Delta f(bq^{j})]$, and we find

$$\int_{a}^{b} \bigl[f(x)D_{q} g(x) + g(q x)D_{q} f(x) \bigr]\,d_{q} x=-f \bigl(bq^{n} \bigr)g \bigl(bq^{n} \bigr). $$

Using the elementary inequality $ab \le\frac{1}{2} ( {a^{2} + b^{2} } )$, and considering that

$$f \bigl(bq^{n} \bigr) =\sum_{j = 0}^{n - 1} \Delta f \bigl(bq^{j} \bigr),\qquad g \bigl(bq^{n} \bigr) = \sum_{j = 0}^{n - 1}\Delta g \bigl(bq^{j} \bigr), $$

by virtue of the Schwarz inequality, we find

$$\begin{aligned} f \bigl(bq^{n} \bigr)g \bigl(bq^{n} \bigr) \le& \frac{1}{2} \Biggl[ \Biggl(\sum_{j = 0}^{n - 1} \Delta f \bigl(bq^{j} \bigr) \Biggr)^{2} + \Biggl(\sum _{j = 0}^{n - 1} \Delta g \bigl(bq^{j} \bigr) \Biggr)^{2} \Biggr] \\ =& \frac{n^{2}}{2} \Biggl[ \Biggl(\frac{1}{n}\sum _{j = 0}^{n -1} {\Delta f \bigl(bq^{j} \bigr)} \Biggr)^{2}+ \Biggl(\frac{1}{n}\sum_{j =0}^{n - 1} {\Delta g \bigl(bq^{j} \bigr)} \Biggr)^{2} \Biggr] \\ \le&\frac{n}{2}\sum_{j = 0}^{n - 1} { \bigl[ \bigl(\Delta f \bigl(bq^{j} \bigr) \bigr)^{2} + \bigl( \Delta g \bigl(bq^{j} \bigr) \bigr)^{2} \bigr]}, \end{aligned}$$

whence, because *f* and *g* are *q*-decreasing functions, we obtain the inequality

$$\begin{aligned} \int_{a}^{b} \bigl[{f(x)D_{q} g(x)+ g(q x)D_{q} f(x)} \bigr]\,d_{q} x \le& -\frac{n}{2}\sum _{j = 0}^{n - 1} \bigl[ \bigl(\Delta f \bigl(bq^{j} \bigr) \bigr)^{2} + \bigl(\Delta g \bigl(bq^{j} \bigr) \bigr)^{2} \bigr] \\ =& -\frac{nb}{2}(1 - q) \int_{a}^{b} \bigl[ \bigl(D_{q} f(x) \bigr)^{2} + \bigl(D_{q} g(x) \bigr)^{2} \bigr]\,d_{q} x. \end{aligned}$$

However, since $n \ge[n]_{q} = \frac{{1 - q^{n} }}{{1 - q}}$, there follows $-n(1 - q) \le q^{n}-1$, so we have

$$\begin{aligned} \int_{a}^{b} \bigl[f(x)D_{q} g(x)+g(q x)D_{q} f(x) \bigr]\,d_{q} x \le& \frac{b}{2} \bigl(q^{n} - 1 \bigr) \int_{a}^{b} \bigl[(D_{q} f(x)^{2} + ( D_{q} g(x)^{2} \bigr]\,d_{q} x \\ =&\frac{a - b}{2} \int_{a}^{b} \bigl[ \bigl(D_{q} f(x) \bigr)^{2} + \bigl(D_{q} g(x) \bigr)^{2} \bigr]\,d_{q} x. \end{aligned}$$

Thereby (6) is proved. □

### Theorem 3.4

*If* $f(x)$ *and* $g(x)$ *are absolutely continuous* *q*-*decreasing functions on* $( {a,b} )$ *and satisfy* $f(bq^{0}) = f(bq^{n}) = 0$, $g(bq^{0}) = g(bq^{n}) = 0$, *then we have the inequality*

7 $$ \int_{a}^{b} \bigl\vert f(x) \bigr\vert ^{s} \bigl\vert g(x) \bigr\vert ^{t}\, d_{q} x \le\frac{(\frac{b-a}{2})^{s+t}}{s+t} \biggl(s \int_{a}^{b} \bigl(D_{q} f(x) \bigr)^{s + t}\, d_{q} x +t \int_{a}^{b} \bigl(D_{q} g(x) \bigr)^{s+t}\,d_{q} x \biggr). $$

### Proof

For $k \in N_{0}$, we have the following identities:

8 $$\begin{aligned} &f \bigl(bq^{k} \bigr)=\sum_{i = 0}^{k - 1} {\Delta f \bigl(bq^{i} \bigr)},\qquad f \bigl(bq^{k} \bigr) =- \sum_{i=k}^{n - 1}\Delta f \bigl(bq^{i} \bigr) , \end{aligned}$$

9 $$\begin{aligned} &g \bigl(bq^{k} \bigr)=\sum_{i=0}^{n-1} \Delta g \bigl(bq^{i} \bigr),\qquad g \bigl(bq^{k} \bigr)=- \sum_{i = k}^{n - 1}\Delta g \bigl(bq^{i} \bigr) . \end{aligned}$$

From (8) and (9) we observe that

10 $$ \bigl\vert f \bigl(bq^{k} \bigr) \bigr\vert \le \frac{1}{2}\sum_{i = 0}^{n - 1} \bigl\vert \Delta f(bq^{i} \bigr\vert ,\qquad \bigl\vert g \bigl(bq^{k} \bigr) \bigr\vert \le\frac{1}{2}\sum _{i = 0}^{n - 1} \bigl\vert \Delta g \bigl(bq^{i} \bigr) \bigr\vert . $$

From (10) and using the elementary inequality

$$sz^{s + t} + tw^{s + t} - (s + t)z^{s} w^{t} \ge0, $$

where $z,w \ge0$ and $s, t > 0$ are real numbers, we find

11 $$ \bigl\vert f \bigl(bq^{k} \bigr) \bigr\vert ^{s} \bigl\vert g \bigl(bq^{k} \bigr) \bigr\vert ^{t}\le\frac{(\frac{1}{2})^{s+t}}{s+t} \Biggl[s \Biggl(\sum _{i=0}^{n-1} \bigl\vert \Delta f \bigl(bq^{i} \bigr) \bigr\vert \Biggr)^{s+t}+t \Biggl(\sum _{i=0}^{n-1} \bigl\vert \Delta g \bigl(bq^{i} \bigr) \bigr\vert \Biggr)^{s+t} \Biggr]. $$

Using Hölder’s inequality on the right side of (11) with indices $s + t$, $\frac{s + t}{s + t - 1}$, we have

12 $$ \bigl\vert f \bigl(bq^{k} \bigr) \bigr\vert ^{s} \bigl\vert g \bigl(bq^{k} \bigr) \bigr\vert ^{t} \le\frac{(\frac{1}{2})^{s+t}}{s+t} n^{s+t-1} \Biggl(s\sum _{i=0}^{n - 1} \bigl\vert \Delta f \bigl(bq^{i} \bigr) \bigr\vert ^{s + t} +t\sum _{i=0}^{n-1} \bigl\vert \Delta g \bigl(bq^{i} \bigr) \bigr\vert ^{s + t} \Biggr). $$

Summing the inequality (12) from 0 to $n-1$, we obtain

13 $$ \sum_{k = 0}^{n - 1} \bigl\vert f \bigl(bq^{k} \bigr) \bigr\vert ^{s} \bigl\vert g \bigl(bq^{k} \bigr) \bigr\vert ^{t}\le \frac{(\frac{n}{2})^{s + t}}{s+t} \Biggl(s\sum_{k = 0}^{n - 1} \bigl\vert \Delta f \bigl(bq^{k} \bigr) \bigr\vert ^{s + t} +t\sum _{k = 0}^{n - 1} \bigl\vert \Delta g \bigl(bq^{k} \bigr) \bigr\vert ^{s + t} \Biggr). $$

After multiplying the left-hand side of (13) by $b(1 - q)q^{k}$, we transform it into the form of

$$\frac{1}{b}\frac{1}{1-q}\sum_{k=0}^{n-1}b(1 - q)q^{k}\frac{ \vert f(bq^{k}) \vert ^{s} \vert g(bq^{k}) \vert ^{t}}{q^{k}}, $$

and after multiplying the right-hand side of (13) by $(b(1 - q)q^{k})^{s+t-1}$, we transform it into the form of

$$\begin{aligned} &\frac{(\frac{n}{2})^{s + t}}{s + t} \bigl(b(1 - q) \bigr)^{s+t-1} \Biggl(s\sum _{k = 0}^{n - 1} { \bigl(q^{k} \bigr)^{s + t - 1} \frac{ \vert f(bq^{k}) - f(bq^{k + 1}) \vert ^{s + t} }{b^{s + t - 1} (1- q)^{s+ t - 1} (q^{k})^{s + t - 1} }} \\ &\quad {} +t\sum_{k = 0}^{n - 1} \bigl(q^{k} \bigr)^{s + t- 1} \frac{ \vert g(bq^{k}) - g(bq^{k + 1}) \vert ^{s + t} }{b^{s + t - 1} (1 - q)^{s + t - 1} (q^{k})^{s + t - 1} } \Biggr). \end{aligned}$$

Thus we obtain a new form of the inequality (13). Multiplying both sides by $b({1-q})$, we have

$$\begin{aligned} &\sum_{k = 0}^{n - 1} b(1 - q)q^{k} \bigl\vert f \bigl(bq^{k} \bigr) \bigr\vert ^{s} \bigl\vert g \bigl(bq^{k} \bigr) \bigr\vert ^{t} \\ &\quad \le\frac{(\frac{n}{2})^{s + t}}{s + t} \bigl(b(1 - q) \bigr)^{s + t} \Biggl( s \sum_{k = 0}^{n - 1}\frac{ \vert f(bq^{k}) - f(bq^{k + 1}) \vert ^{s + t} }{b^{s + t} (1 - q)^{s + t} (q^{k})^{s + t - 1} } \\ &\qquad{} +t\sum_{k = 0}^{n - 1} \frac{ \vert g(bq^{k}) - g(bq^{k + 1}) \vert ^{s + t} }{b^{s + t - 1} (1 - q)^{s + t - 1} (q^{k})^{s + t - 1} } \Biggr). \end{aligned}$$

Substituting the left-hand side for the corresponding *q*-restricted integral, and the right-hand side for the corresponding *q*-derivatives and *q*-restricted integrals, we obtain

$$\begin{aligned} \int_{bq^{n} }^{b} \bigl\vert f(x) \bigr\vert ^{s} \bigl\vert g(x) \bigr\vert ^{t} \,d_{q} x \le&\frac{(\frac{n}{2})^{s + t}}{s + t} \bigl(b(1 - q) \bigr)^{s + t} \biggl(s \int_{bq^{n}}^{b} \bigl(D_{q} f(x) \bigr)^{s + t}\, d_{q} x \\ &{}+ t \int_{bq^{n}}^{b} \bigl(D_{q} g(x) \bigr)^{s+t} \,d_{q} x \biggr). \end{aligned}$$

After interchanging the boundaries in the right-hand side integrals and multiplying both sides of the last inequality by $b({1-q})$, we obtain

$$\begin{aligned} \int_{bq^{n} }^{b} \bigl\vert f(x) \bigr\vert ^{s} \bigl\vert g(x) \bigr\vert ^{t} \,d_{q} x \le& -\frac{(\frac{n}{2})^{s + t}}{s+t}\, \bigl(b(1 -q) \bigr)^{s + t} \biggl(s \int_{b}^{bq^{n}} \bigl(D_{q} f(x) \bigr)^{s+t} \,d_{q} x \\ &{}+t \int_{b}^{bq^{n}} \bigl(D_{q} g(x) \bigr)^{s + t} \,d_{q} x \biggr). \end{aligned}$$

Since $-(1 - q)^{s + t} n^{s + t} \le -(1 - q^{n})^{s + t}$, we find

$$\begin{aligned} \int_{bq^{n} }^{b} \bigl\vert f(x) \bigr\vert ^{s} \bigl\vert g(x) \bigr\vert ^{t}\, d_{q} x \le&\frac{(\frac{1}{2})^{s + t}}{s + t}\,b^{s+t} \bigl(1 - q^{n} \bigr)^{s + t} \biggl( s \int_{bq^{n} }^{b} \bigl(D_{q} f(x) \bigr)^{s + t} \,d_{q} x \\ &{}+t \int_{bq^{n} }^{b} \bigl(D_{q} g(x) \bigr)^{s + t} \,d_{q} x \biggr), \end{aligned}$$

and we finally arrive at the inequality

$$\int_{a}^{b} \bigl\vert f(x) \bigr\vert ^{s} \bigl\vert g(x) \bigr\vert ^{t}\, d_{q} x \le \frac{(\frac{{b - a}}{2})^{s + t}}{s+t} \biggl[s \int_{a}^{b} \bigl(D_{q} f(x) \bigr)^{s + t} \,d_{q} x +t \int_{a}^{b} \bigl(D_{q} g(x) \bigr)^{s + t}\,d_{q} x \biggr], $$

whereby we complete the proof. □

### Remark 3.5

We note that, in the special case when $s = t = r$ and $f(x) = g(x) = h(x)$, the inequality established in (7) reduces to the following *q*-Wirtinger inequality:

$$\int_{a}^{b} { \bigl\vert h(x) \bigr\vert ^{2r} \,d_{q} x} \le \biggl(\frac{b - a}{2} \biggr)^{2r} \int_{a}^{b} { \bigl(D_{q} h(x) \bigr)^{2r}\, d_{q} x}. $$

## Conclusions

In this paper we have established a new general Opial type integral inequality in *q*-calculus. Further, we investigated the Opial inequalities in *q*-calculus involving two functions and their first order derivatives. We also discussed several particular cases. The method we used to establish our results is quite elementary and based on some simple observations and applications of some fundamental inequalities.
